# Perovskite SrZrO_3_:Ho^3+^ phosphors: synthesis, structure, Judd–Ofelt analysis and photoluminescence properties

**DOI:** 10.1039/d3ra04175a

**Published:** 2023-09-19

**Authors:** Vijay Singh, M. Seshadri

**Affiliations:** a Department of Chemical Engineering, Konkuk University Seoul 05029 Republic of Korea vijayjiin2006@yahoo.com; b Department of Physics, Koneru Lakshmaiah Education Foundation Hyderabad 500043 Telangana India

## Abstract

A series of SrZrO_3_:*x*Ho^3+^ (*x* = 0.01, 0.03, 0.05, 0.07, 0.09, and 0.11 mol) perovskite phosphors have been synthesized by using the sol–gel technique. The structural and optical characteristics of the prepared phosphors have been investigated through powder XRD, FT-IR, UV-visible diffuse reflectance, and photoluminescence analysis. The photoluminescence emission spectra showed a bright characteristic peak at 545 nm (^5^F_4_ + ^5^S_2_ → ^5^I_8_) under the 454 nm excitation, which exhibits emission in the green region of the electromagnetic spectrum. The emission intensity of the phosphors starts decreasing slowly beyond 3 mol% Ho^3+^ ions concentration due to concentration quenching, which is attributed to the dipole–dipole interaction between Ho^3+^ ions. The site symmetry of the Ho^3+^ ions has been studied by estimating the relative Judd–Ofelt intensity parameters (*Ω*_*λ*_, where *λ* = 2, 4, 6) from the photoluminescence excitation spectrum of the SrZrO_3_:0.03Ho^3+^ phosphor. The obtained findings suggest that the synthesized phosphors will be favorable for their bright green emission and thus, can be widely used for different optoelectronic applications.

## Abstract 2

A series of SrZrO_3_:*x*Ho^3+^ (*x* = 0.01, 0.03, 0.05, 0.07, 0.09, and 0.11 mol) perovskite phosphors have been synthesized by using the sol–gel technique.

## Graphical abstract

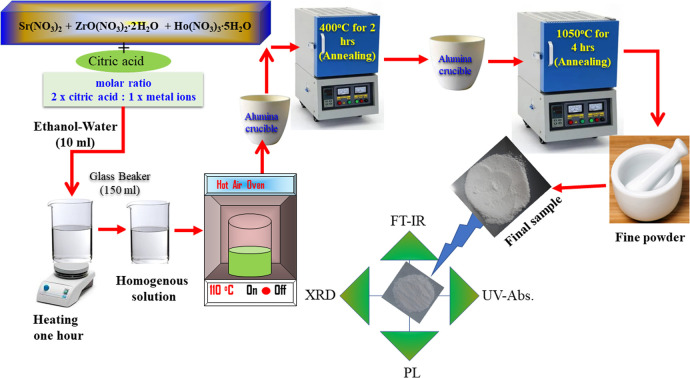

## 1. Introduction

Perovskite materials with the general formula ABO_3_ (where A = Ca, Sr, Ba; and B = Zr, Hf, Ti) have great versatility and outstanding chemical, physical, electrical, and thermo-mechanical properties. These host lattices are getting special attention due to their potential applications as catalysts, photocatalysts, fuel cells, in photovoltaic applications, optoelectronics, and solid oxide fuel cells.^1–3^ Perovskite materials possess a fascinating feature in which a slight variation in structure and chemical composition may result in enormous changes in their chemical and physical properties.^4–7^ The doping of a foreign element into these ABO_3_ type inorganic oxides influences their optical and magnetic properties by creating various defects.^8,9^ Especially, lanthanide ions as dopants are suitable candidates since they exhibit unique spectroscopic properties.^10–12^ The coordination geometry and oxidation states of uranium ions in the SrZrO_3_ perovskite were studied by Gupta *et al.*,^13^ although Li *et al.*^14^ investigated the spectral characteristics and intrinsic defects of SrZrO_3_ perovskite. The earlier report published by Knight *et al.*^15^ reveals the structural and thermoelastic characteristics of SrZrO_3_ perovskite.

Perovskites have been reported to be advantageous in high-temperature applications, like hydrogen gas sensors, steam electrolysis, and fuel cells.^16–18^ Proton conductivity at elevated temperature enables its usage in typical electrochemical devices. Among different perovskite materials, the SrZrO_3_ host material has been suggested for use as a potential substrate due to its large single crystals.^19^ In recent years, the structural phase transitions of strontium zirconate perovskites have been studied significantly at room temperature and higher temperatures.^20–22^ Initially, the structural investigations revealed that the SrZrO_3_ possesses an orthorhombic phase at room temperature, and later Carlsson^23^ proposed the presence of additional phases at high temperatures. Mete *et al.*^17^ examined the structural and electronic characteristics of 4d-perovskite: the cubic phase of SrZrO_3_. The electronic and structural performances of selected surfaces of SrZrO_3_ were investigated by Sambrano *et al.*^24^ Singh *et al.*^25^ have also published research on the photoluminescence and structural properties of SrZrO_3_:Sm^3+^ orange-emitting perovskite phosphors.

Due to long-lived excited states and energy levels, the trivalent lanthanide ions doped luminescent materials (using Tm^3+^, Er^3+^, and Ho^3+^ ions) have been selected as the main subject of interest by several research groups.^26–28^ Holmium belongs to the lanthanides and its electronic configuration becomes [Xe]4f^10^ when doped into a crystalline host. Several spectroscopic studies revealed that Ho^3+^ is the most desirable ion for mid-infrared lasers and has excellent green emission properties other than Tb^3+^ (green emission only) ions among the rare earth ions due to its various electronic transitions. Ranjan *et al.*^29^ reported the enhanced green up-conversion emission of Ho^3+^ doped Gd_2_O_3_ phosphor by co-doping with Yb^3+^ ions. The luminescence studies of Eu^3+^ and Ho^3+^ doped Sr_2_TiO_4_ revealed that the Sr_2_TiO_4_ could be a suitable material in favor of high-pressure mercury vapor lamps or white light-emitting diodes.^30^

A literature survey confirms a lack of reports available on Ho^3+^ doped ABO_3_ type zirconate perovskites. The Ho^3+^ is often used as a structural probe because it can be accommodated at the A-place or B-place of perovskite oxides and in-site changes in the optical behavior and local site surrounding these doped oxide materials. Based on these results, our prepared sample possesses excellent thermal and chemical stability, prompting its practical application. Combining trivalent rare-earth metal ions in zirconate perovskites is considered as a promising approach to developing more useful and stable luminescent materials. Shi *et al.*^31^ prepared Yb^3+^, Ho^3+^, Li^+^ tri-doped TiO_2_ up-conversion materials to enhance the efficiency of perovskite solar cells. First, Hou *et al.*^32^ investigated the impacts of Ho^3+^ ion doping over the surface morphology, crystal phase, and magnetic characteristics of BiFeO_3_ thin films synthesized by the sol–gel technique. Sharif *et al.*^33^ investigated surface morphology, structural, magnetic, and dielectric properties in the BiFeO_3_ with holmium-doped thin films deposited by the pulsed laser deposition technique. Moreover, Hussain *et al.*^34^ presented resistive leakage and intrinsic polarization analyses for high-performance piezo/pyroelectric Ho-doped 0.64Pb(Mg_1/3_Nb_2/3_)O_3_-0.36PbTiO_3_ binary ceramic materials.

Besides the solid-state reaction process, a conventional synthetic route for preparing phosphor particles, several new synthetic methods have already been developed, for example, co-precipitation, sol–gel, solution combustion, microemulsion, spray pyrolysis, and hydrothermal synthesis. The present work uses the sol–gel method to prepare SrZrO_3_ phosphor. Sol–gel is a synthetic route to synthesize ceramic oxides, which provides reasonable control over stoichiometry, high purity, good homogeneity, and reduced sintering temperature. This method may also enable the production of low-temperature phases. A sol–gel method in a liquid includes a polycondensation reaction, which builds the oxide network of a molecular precursor. Although the process consists of several steps, doping concentration positively impacts the luminescence and crystal structural properties of the prepared phosphor. Wurm *et al.*^35^ prepared sol–gel SrZrO_3_ and SrTiO_3_ coatings on C and SiC-fibers. Venkatesh *et al.*^36^ prepared a novel strontium zirconate perovskite coating on an Inconel substrate using the sol–gel synthesis method. Liu *et al.*^37^ reported sol–gel derived SrZrO_3_ memory thin films with resistance switching properties. In this work, the Ho^3+^ doped SrZrO_3_ perovskite phosphors were synthesized by using sol–gel synthesis. The prepared phosphors were characterized structurally and optically. To know the spectral characteristics, the measured photoluminescence excitation spectra were used to calculate the Judd–Ofelt intensity parameters, *Ω*_2_, *Ω*_4_ & *Ω*_6_. Detailed photoluminescence properties are discussed herein.

## 2. Materials preparation and analysis

**Fig. 1:**
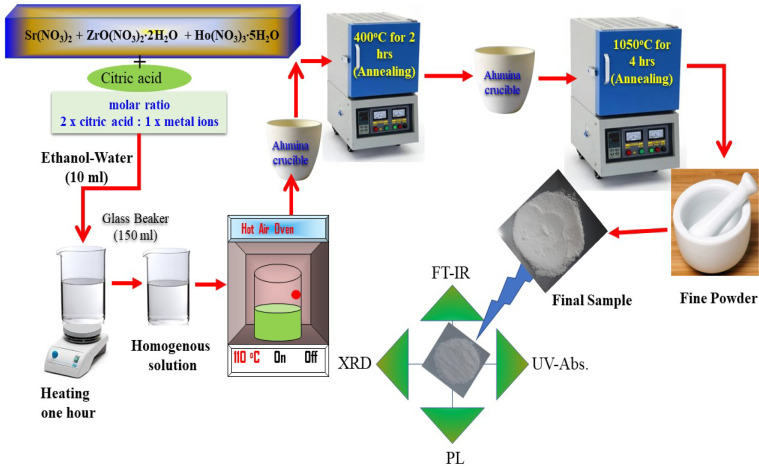
Systematic diagram of synthesis process of the sample.

The SrZrO_3_:*x*Ho^3+^ (*x* = 0.01, 0.03, 0.05, 0.07, 0.09, and 0.11 mol) perovskite phosphors were fabricated by sol–gel procedure. The quantity of the employed starting materials is reported in Table 1. As per the chemical formulae, the stoichiometric quantities of strontium nitrate (Sr(NO_3_)_2_) (Sigma-Aldrich, purity: 99%), zirconium nitrate oxide dihydrate (ZrO(NO_3_)_2_·2H_2_O) (Kanto chemical, purity: 99%), holmium(iii) nitrate pentahydrate (Ho(NO_3_)_3_·5H_2_O) (Sigma-Aldrich, purity: 99.9%), citric acid (C_6_H_8_O_7_) (Junsei, purity: 99.5%) and, a mixture of 6 ml of ethanol and 4 ml of water combine in a 150 ml beaker. The molar ratio was kept at 2 : 1 according to citric acid and total metal ions. Next, the mixture was stirred for 1 h to achieve a clear homogeneous solution; after that, put the resultant in the oven until the solution dried. A hot temperature furnace maintained at 400 °C preheated the acquired gels for 2 h in air. After preheating, the samples were granulated and fired for 4 h at 1050 °C in the ambient condition, giving the fine powder samples. Fig. 1 presents a pictorial view of the synthesis process.

**Table 1:** Detailed information of sample composition and starting materials

Sample compositions	Weight of starting materials (g)			
	Sr(NO_3_)_2_	ZrO(NO_3_)_2_·2H_2_O	C_6_H_8_O_7_	Ho(NO_3_)_3_·5H_2_O
SrZrO_3_:0.01Ho	0.8464	1.0688	3.0738	0.0176
SrZrO_3_:0.03Ho	0.8464	1.0688	3.0738	0.0592
SrZrO_3_:0.05Ho	0.8464	1.0688	3.0738	0.0882
SrZrO_3_:0.07Ho	0.8464	1.0688	3.0738	0.1243
SrZrO_3_:.0.09Ho	0.8464	1.0688	3.0738	0.1578
SrZrO_3_:0.11Ho	0.8464	1.0688	3.0738	0.1940

The X-ray diffraction patterns were monitored by a RIGAKU (Miniflex-II) diffractometer attached to an X-ray source (Cu-Kα radiation, *λ* = 1.5406 Å); the scan rate was set at 5° per minute between 10° −80° for 2*θ* angle. To identify the functional group present in the prepared samples, a Fourier transform infrared (6700, Thermo Fisher Nicolet) spectrometer was operated in a range of 400–4000 cm^−1^. A small quantity of prepared SrZrO_3_:Ho^3+^ phosphor powder is used to measure diffuse reflectance with A Cary-5000 (UV-VIS-NIR) spectrophotometer coupled to a Praying Mantis diffuse reflectance accessory. Photoluminescence (PL) spectra were analyzed by a Shimadzu (RF-5301PC) spectrofluorophotometer fitted with a Xenon-flash lamp. The emission and excitation spectra were recorded using a spectral slit width of 1.5 nm. The above characterizations were performed at room temperature.

## 3. Results and discussion

### 3.1 Crystal structure

**Fig. 2:**
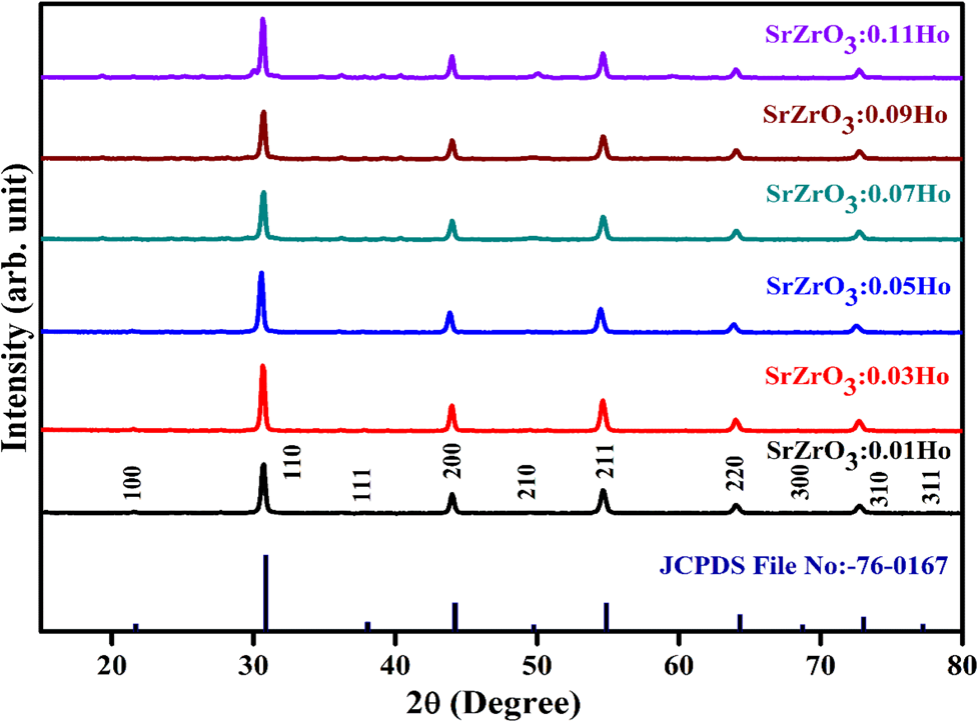
Powder XRD patterns of SrZrO_3_:*x*Ho^3+^ phosphors.

**Fig. 3:**
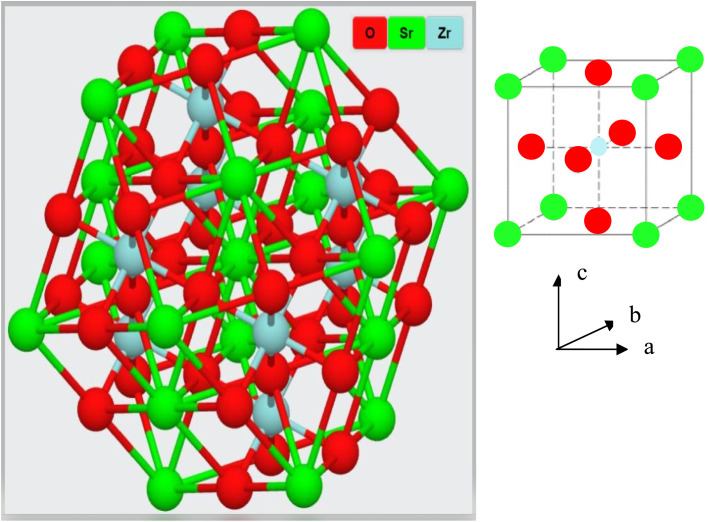
Crystal structure of the SrZrO_3_ perovskite.

The XRD measurement was conducted to study the structural phase and crystallinity of the prepared samples. Fig. 2 displays the XRD patterns of the SrZrO_3_:*x*Ho^3+^ powders. For the synthesized phosphors, the major diffraction peaks matched with JCPDS (Joint Committee for Powder Diffraction Standards Card) File No. 76-0167 corresponding to SrZrO_3_. The effect of Ho^3+^ ions (at the studied concentrations *i.e.* 0.01, 0.03, 0.05, 0.07, 0.09, and 0.11 mol) on the structure of the SrZrO_3_ lattice seems to be negligible as XRD patterns remain the almost same at different doping concentrations. However, we believe the actual doping level can be clarified by Le Bail method to find the evolution trend of the cell lattice parameters. To calculate the crystalline size, the FWHM (full width at half maximum) of dominant (110) diffraction peaks are considered in leading Scherrer's equation, *D* = 0.9*λ*/*β* cos *θ*, in which the wavelength of incident X-rays is denoted as *λ*, the corresponding Bragg's diffraction angle is *θ*, and the FWHM of the (110) peak is *β*. The determined crystal sizes were approximately within the range of 24–31 nm. Various oxides with the ABO_3_ chemical formula follow the perovskite structure. Fig. 3 showed the simplified crystal lattice of the SrZrO_3_ perovskite. The SrZrO_3_ has cubic symmetry with a *Pm*3̄*m*[221] space group. In the SrZrO_3_ perovskite structure, the Sr atoms are situated on the edges of the cubic unit cell, and the 12 closest neighbor O atoms surround the Sr atoms. Similarly, the Zr atom is situated in the centrum of the unit cell and is six-fold integrated with the O closed-neighbor atoms, making an octahedron. The cubic unit cell faces have O atoms, which are two-fold coordinated with Zr neighbor atoms. The Zr-ion and Sr-ion have the coordination numbers 6 and 8, respectively.^1,11^

### 3.2 Vibrational analysis

**Fig. 4:**
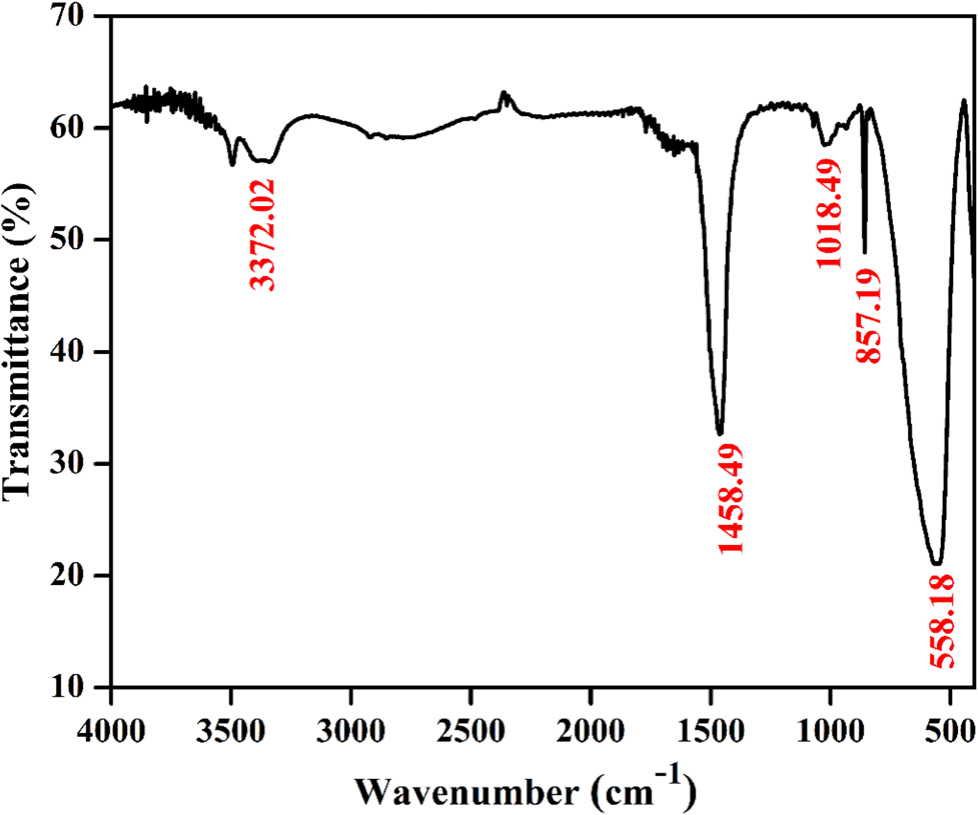
FTIR spectrum of SrZrO_3_:0.03Ho^3+^ phosphor.

Fig. 4 shows a typical vibrational feature of the SrZrO_3_:0.03Ho^3+^ powder sample. An intense absorption band at 558 cm^−1^ is attributed to the Zr–O stretching vibration. We have also observed a sharp peak at 857 cm^−1^. Katyayan and Agrawal^8^ studied SrZrO_3_:Eu^3+^, Tb^3+^ system and several peaks reported in the range of 509–895 cm^−1^ were due to the vibrational stretching modes of metal–oxygen bond, *i.e.*, Zr–O bond. However, few peaks lie between 1000 and 1270 cm^−1^ due to the active modes of asymmetric stretching of impurity ions. Further, sharp peaks were reported within 1302–1588 cm^−1^ due to the symmetrical stretching of Sr–O bonds. We have also observed bands at 1018 and 1458 cm^−1^. The observed bands and their assignments can be confirmed with the previous literature.^8,38–41^

### 3.3 Diffuse reflectance spectra and optical band gap

**Fig. 5:**
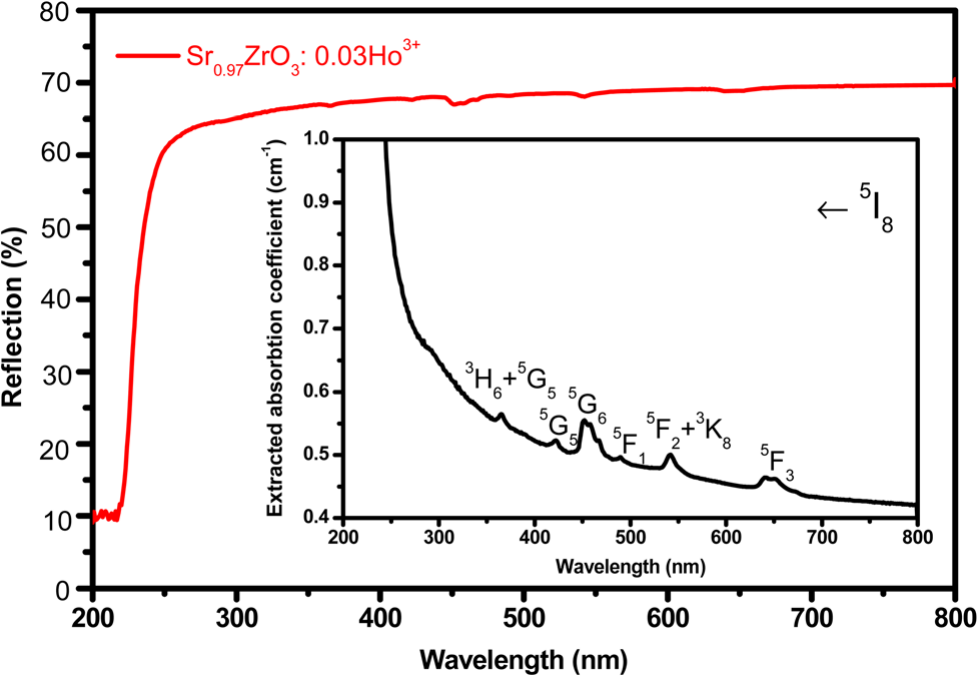
UV-visible diffuse reflectance spectrum of SrZrO_3_:0.03Ho^3+^ phosphor. Inset of the figure shows extracted absorption spectrum.

The UV-diffuse reflectance spectra were recorded between the wavelength regions of 200–800 nm for the 3 mol% Ho^3+^ doped SrZrO_3_ phosphor. Fig. 5 shows the diffuse reflectance spectrum and extracted absorption coefficient with the Kubelka–Munk function. It can be seen that few bands around 365, 421, 454, 468, 489, 542, and 645 nm are related to the 4f–4f configuration of Ho^3+^ transitions: ^5^I_8_ → ^5^G_5_ + ^3^H_6_, ^5^I_8_ → ^5^G_5_, ^5^I_8_ → ^5^G_6_, ^5^I_8_ → ^5^F_1_, ^5^I_8_ → ^5^F_2_ + ^3^K_8_, and ^5^I_8_ → ^5^F_3_, respectively. The extracted optical-absorption coefficient (*α* cm^−1^) was calculated by the consecutive expression:^42^1
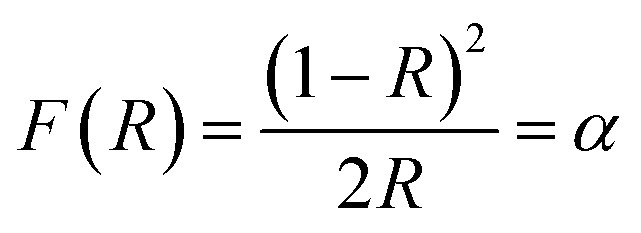
where *R* stands for sample reflectance. The *E*_g_ (optical band gaps) for the Ho^3+^ doped SrZrO_3_ phosphor can be estimated with the Tauc relation:^43^

**Fig. 6:**
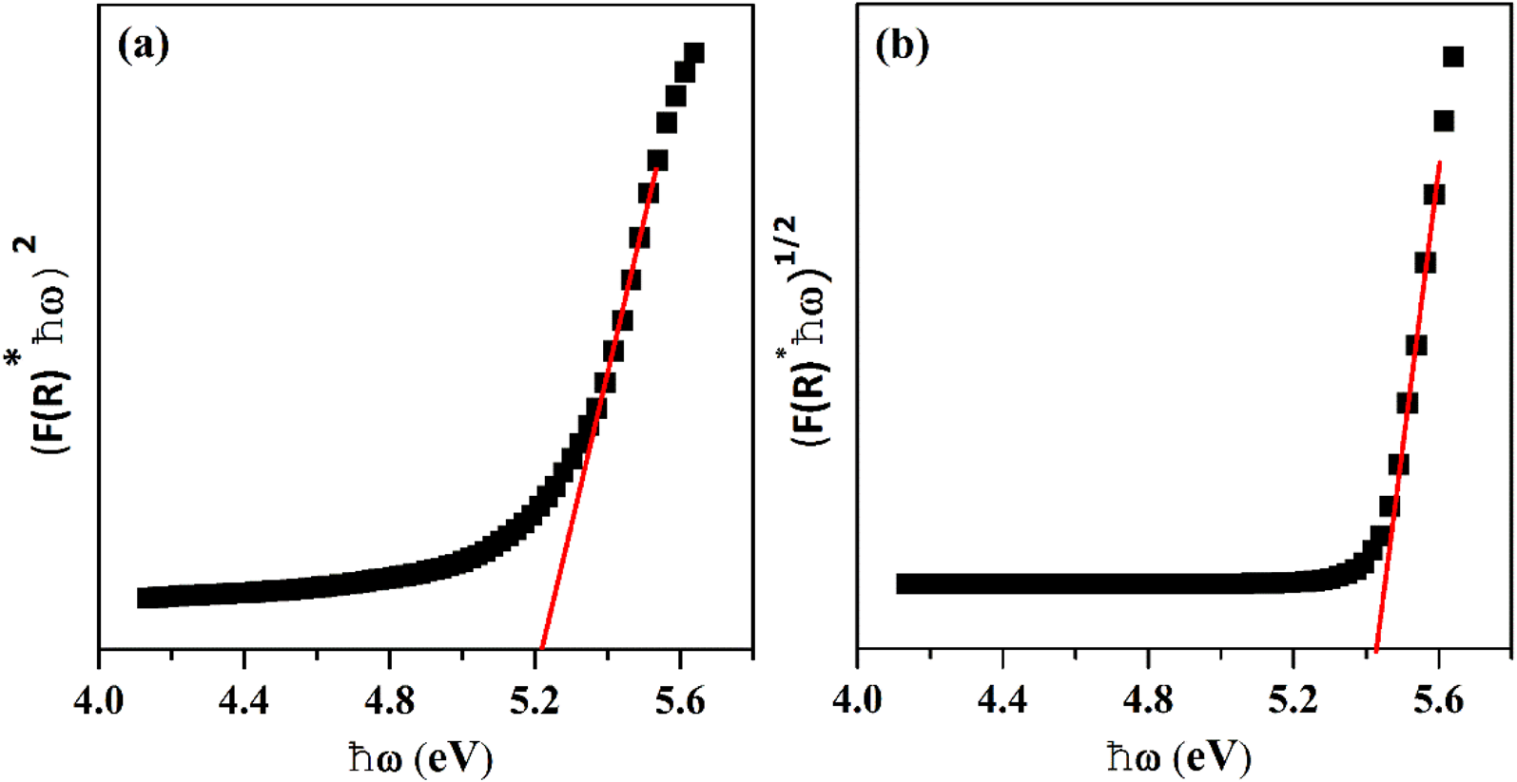
Plots of (*F*(*R*)ℏ*ω*)^*n*^*versus* ℏ*ω* (eV): (a) direct allowed transition and (b) indirect allowed transition.

2 *F*(*R*)ℏ*ω* ≈ *A*(ℏ*ω* − *E*_g_)^*n*^

where *A* is a constant, *F*(*R*) is the absorption coefficient with photon energy (ℏ*ω*) and *n* represents the power factor: *n* = 1/2 allowed direct transitions, and *n* = 2 allowed an indirect transition. Fig. 6 shows plots of (*F*(*R*)ℏ*ω*)^*n*^ as a function of ℏ*ω* (eV). After assuming (*F*(*R*)ℏ*ω*)^2^ = 0 and (*F*(*R*)ℏ*ω*)^1/2^ = 0 for the linear region within the plot, the energy band gaps of the direct allowed and indirectly allowed transitions were observed to be 5.21 eV and 5.43 eV, correspondingly for the SrZrO_3_:0.03Ho^3+^ phosphor.

### 3.4 Photoluminescence analysis

**Fig. 7:**
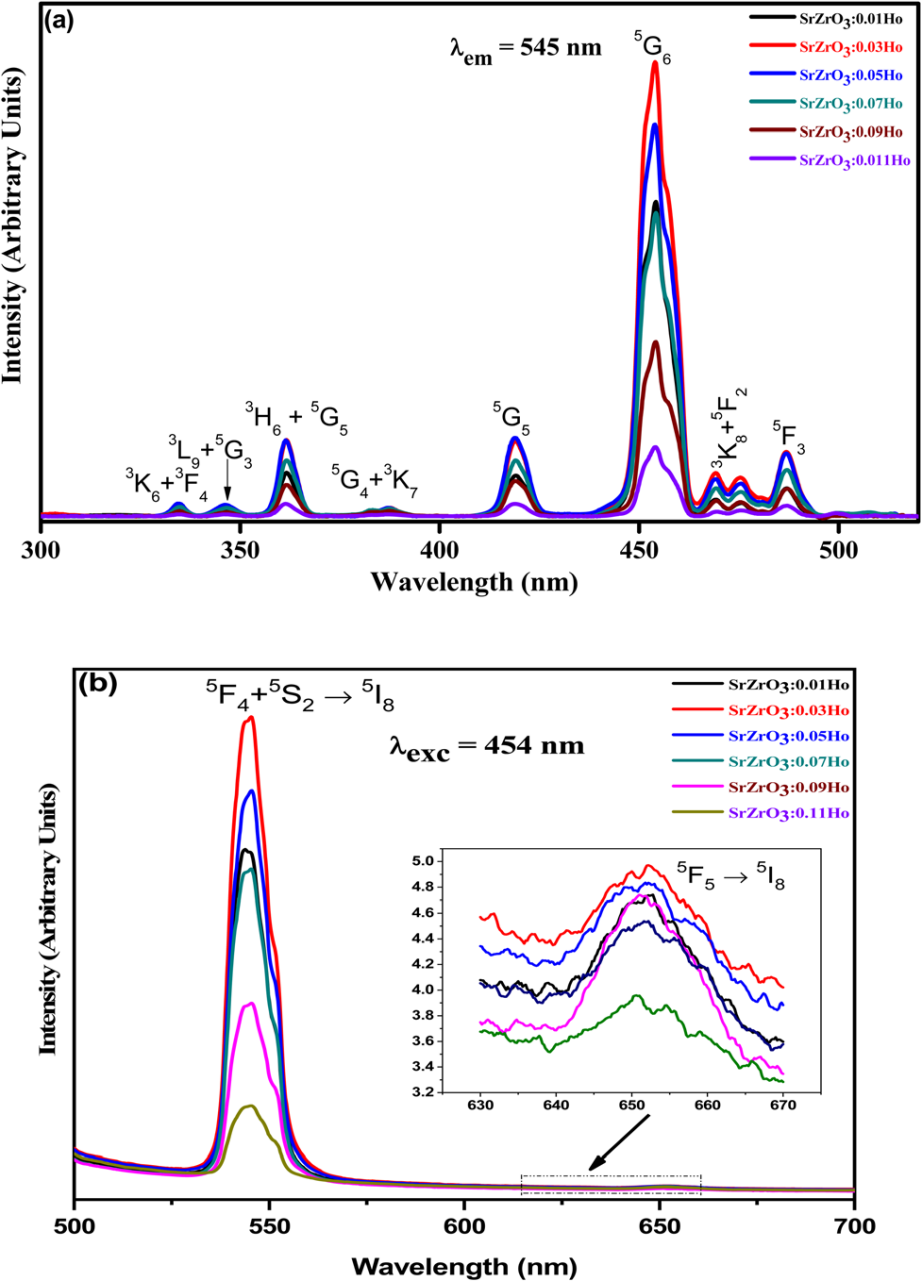
(a) Photoluminescence excitation spectra of SrZrO_3_:*x*Ho^3+^ phosphors (*λ*_em_ = 545 nm) and (b) photoluminescence emission spectra of SrZrO_3_:*x*Ho^3+^ phosphors (*λ*_exc_ = 454 nm).

Fig. 7(a) demonstrates the photoluminescence excitation (PLE) spectra for the produced Ho^3+^ doped SrZrO_3_ phosphors. The PLE spectra were acquired using the emission band at 545 nm. It can be read that the narrow bands owing to the 4f–4f transitions of Ho^3+^ ions and originated from ^5^I_8_ to ^3^K_6_ + ^3^F_4_ (334 nm), ^3^L_9_ + ^5^G_3_ (346 nm), ^3^H_6_ + ^5^G_5_ (361 nm), ^3^K_7_ + ^5^G_4_ (387 nm), ^5^G_5_ (418 nm), ^5^G_6_ (454 nm), ^5^F_2_ + ^3^K_8_ (472 nm) and ^5^F_3_ (486 nm). The highest PLE intensity was observed at 454 nm; this would be suitable as the excitation wavelength for all prepared phosphors. The PLE band intensity is saturated at 3 mol% of Ho^3+^ ions in the SrZrO_3_ phosphor. Usually, the doping of Ho^3+^ ions in place of Sr^2+^ ions will create positive charge defects that would negatively affect luminescence. Therefore, the expected emission intensity is maximum for 3 mol% of Ho^3+^ ions, and then the subsequent decrease of emission intensity is due to the Ho_Sr_^+^ defects.^44^

**Fig. 8:**
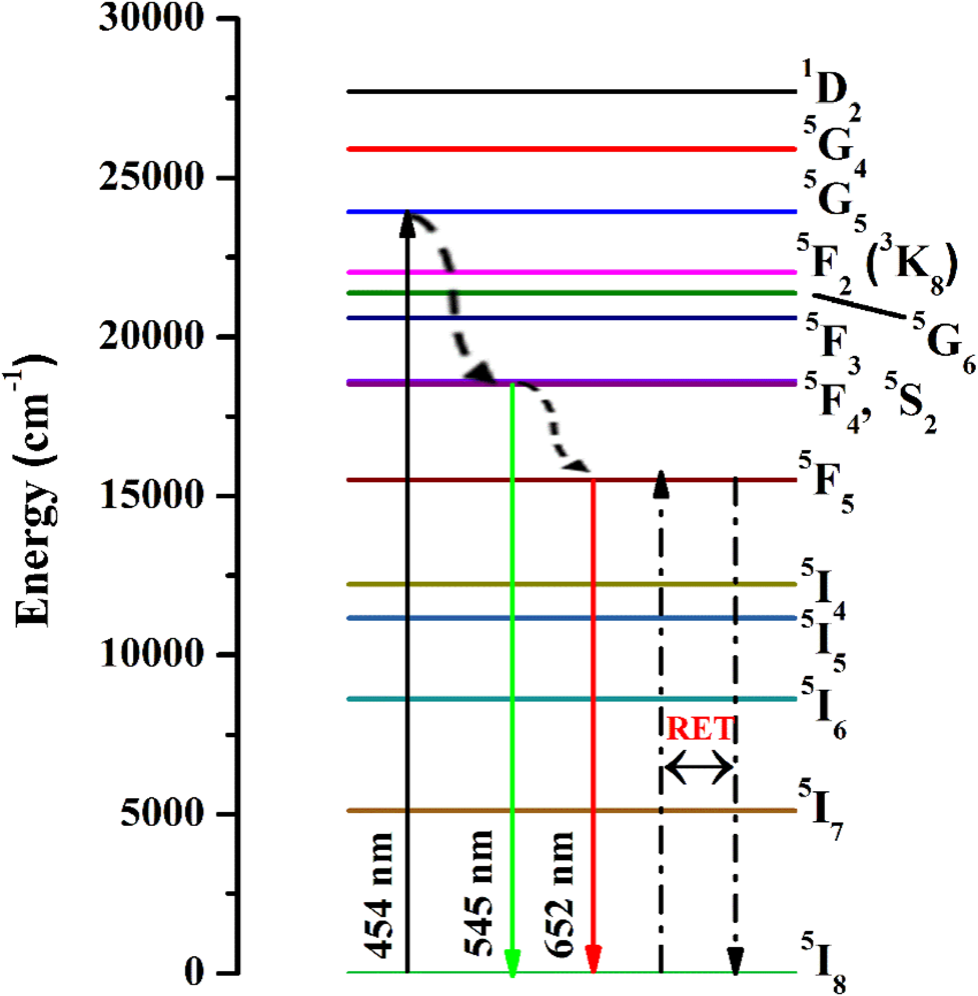
Schematic energy level diagram for Ho^3+^ ions with possible radiative and non-radiative transitions.

The emission spectra were monitored using 454 nm as the excitation wavelength for all phosphors and are presented in Fig. 7(b). Strong green and weak red emission bands have been seen around 545 nm and 652 nm. The obtained bands are possibly defined as transitions of Ho^3+^ ions for ^5^F_4_ + ^5^S_2_ → ^5^I_8_ and ^5^F_5_ → ^5^I_8_, respectively. The intensity of green emission at 545 nm is ∼35 times more substantial than the red emission at 652 nm. Upon excitation of 454 nm, the ions are excited from ^5^I_8_ to ^5^G_4_, then most of the excited ions decay the lower levels ^5^F_4_ + ^5^S_2_ and ^5^F_5_ levels non-radiatively, and subsequently, radiatively transit to ^5^I_8_ level with emission of green (^5^F_4_ + ^5^S_2_ → ^5^I_8_) and red (^5^F_5_ → ^5^I_8_), respectively. The energy gap difference for the ^5^F_4_ and ^5^F_5_ to the next lower levels are around 3400 cm^−1^ and 2400 cm^−1^, thus the observed difference of green and red emission intensities depends on the population of excited states and multiphonon relaxation rates (*W*_mpr_) since the *W*_mpr_ is increasing with the decrease of the energy gap between excited state to the next lower state. Fig. 8 shows a schematic energy level diagram for Ho^3+^ ions with possible radiative and non-radiative relaxation processes.

**Fig. 9:**
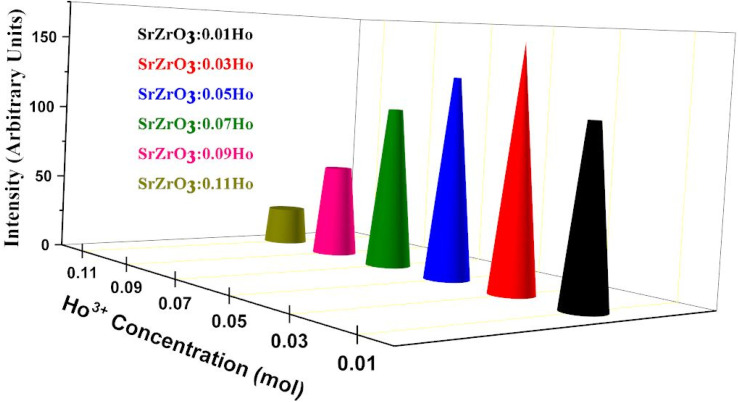
Variation of emission intensity (*I*_545 nm_) as a function of Ho^3+^ ions in the SrZrO_3_:Ho^3+^ phosphor.

**Fig. 10:**
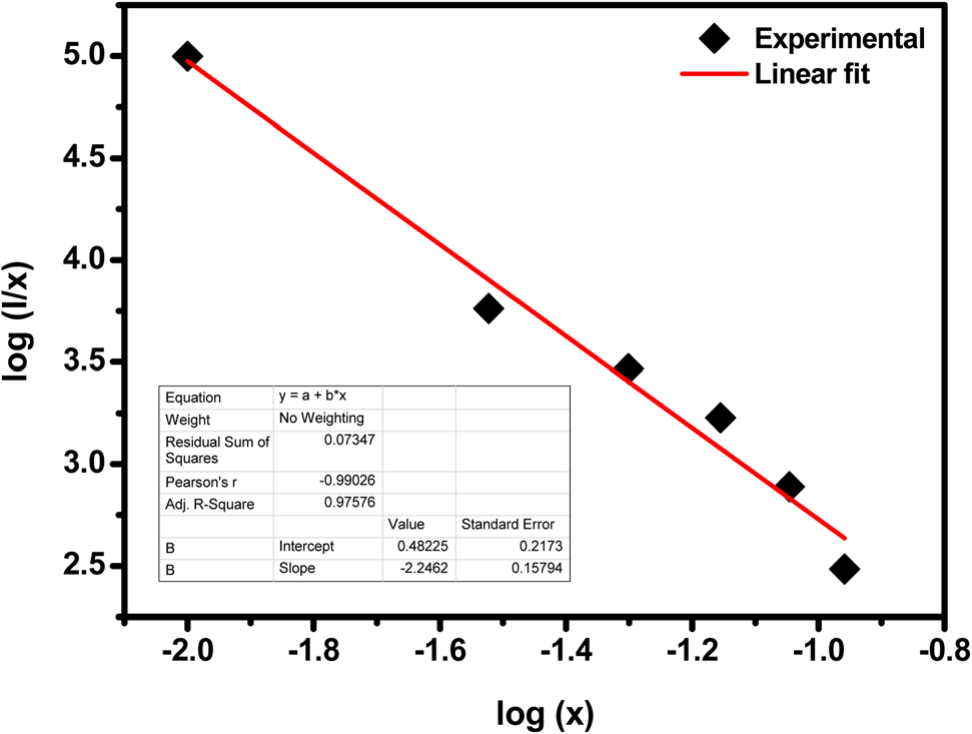
Plot of log(*I*/*x*) as a function of log(*x*) of the SrZrO_3_:0.03Ho^3+^ phosphor (where *I* is the green emission peak intensity, and *x* is the Ho^3+^ ion concentration).

As seen in Fig. 9, the highest emission intensity was noticed when the Ho^3+^ ions concentration was 3 mol%, and it was decreased according to the increasing concentration of Ho^3+^ ions in the SrZrO_3_ phosphor because of concentration quenching. Generally, it is recognized that the Ho–Ho distance reduces with an enhancement of Ho^3+^ ion concentration, leading to fluorescence quenching that comes from an increase in the resonant transfer probability between Ho^3+^ ions. The chances of energy transmission distance between Ho–Ho ions are termed the critical distance (*R*_c_) obtained by the Blasse expression:^45^3
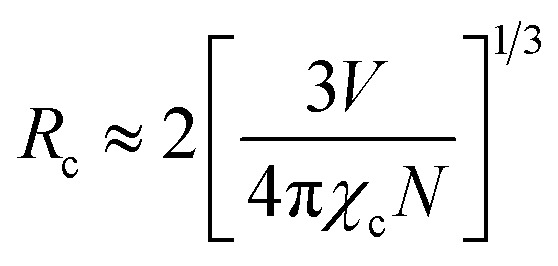
where *V* is the unit cell volume, critical ion concentration is *χ*_c_ and *N* is the number of Zr ions of a unit cell. For our Ho^3+^ doped SrZrO_3_ phosphor, *V* = 552.175 (Å)^3^,^46^*N* = 4, and *χ*_c_ = 0.03. The calculated critical transfer distance (*R*_c_) is ∼20 Å, far greater than 5 Å that favors exchange interaction; thus, it can be established that the observed concentration quenching in Ho^3+^ doped SrZrO_3_ samples is attributed to the multipole–multipoleelectric interaction.^42^ In addition, the interaction strength can also be calculated using the following equation:^47^4
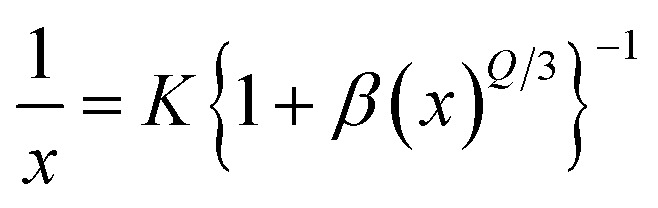
where *K* and *β* are constants, *I* is emission intensity, and *x* is activator ion concentration. The dipole–dipole (d–d), dipole–quadruple (d–q), quadruple–quadruple (q–q) interactions take place with *Q* = 6, 8, and 10, respectively. Fig. 10 shows the log(*I/x*) based on log(*x*) for the SrZrO_3_:0.03Ho^3+^ phosphor. It can be observed that −*Q*/3 = - 2.24, so *Q* = 6.72. Thus, the quenching in emission intensity of Ho^3+^ doped SrZrO_3_ host lattices is due to dipole–dipole interactions. The concentration quenching of Ho^3+^ doped SrZrO_3_ phosphor that occurred beyond the optimized concentration (3 mol%) is most useful for emitting green light in optoelectronic devices.

**Fig. 11:**
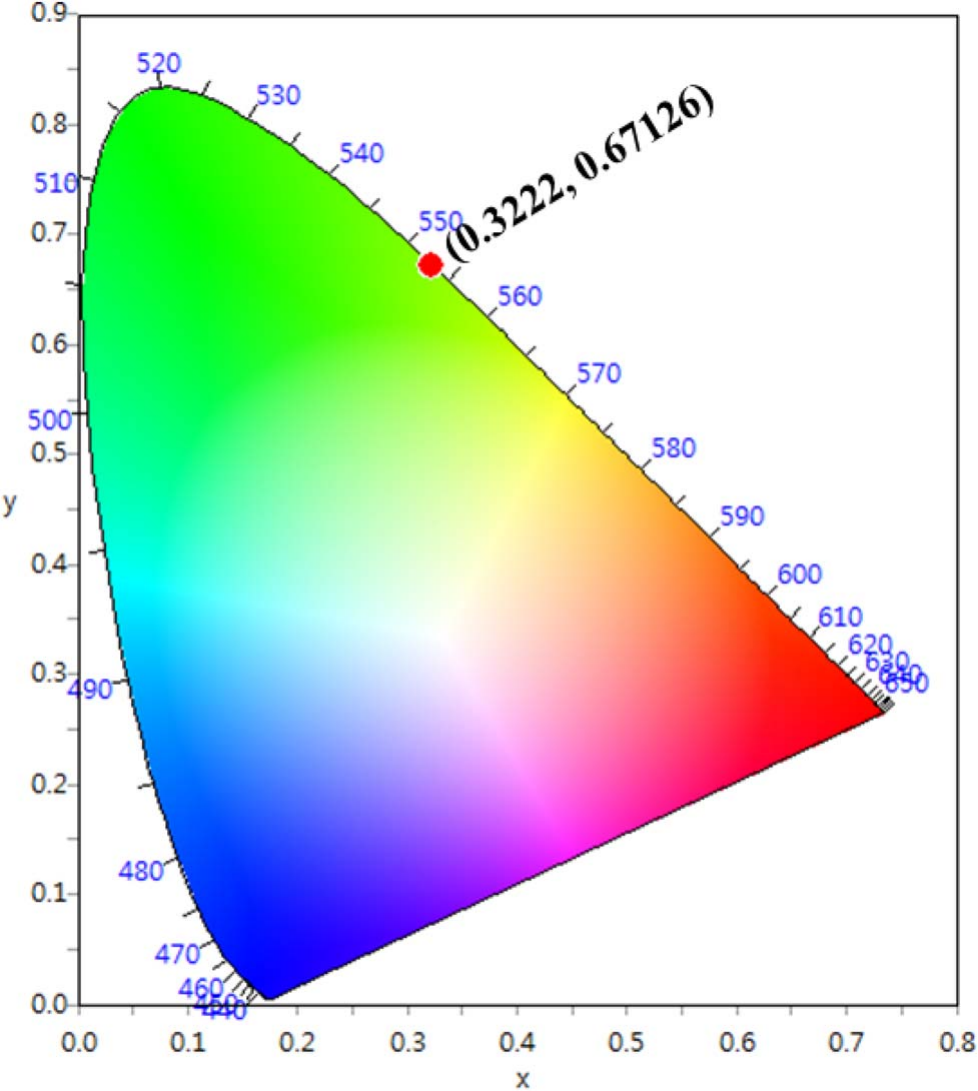
CIE chromaticity diagram of SrZrO_3_:0.03Ho^3+^ phosphor.

The CIE chromaticity coordinates were determined by the emission spectrum (*λ*_exc_ = 454 nm) of optimized Ho^3+^ (3 mol%) doped SrZrO_3_ phosphor using 1931 CIE (Commission International de l’Eclairage France) technology, which is an accepted standard for the LED industry in matters related to colors, such as color mixing and color rendering. The chromaticity coordinates were found to be (0.322, 0.671) and this is situated in the green region of the CIE chromaticity diagram (see Fig. 11). The correlated color temperature (CCT) was also estimated by the McCamy experimental equation:^48^

5 CCT = −437*n*^3^ + 3601*n*^2^ − 6861*n* + 5514.31

where 
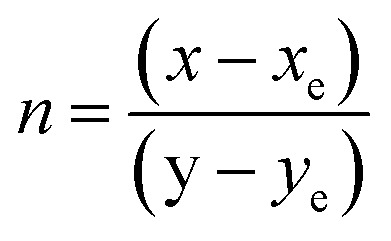
 with chromaticity epicenter being *x*_e_ = 0.3320 and *y*_e_ = 0.1858. The obtained CCT is 5654 K for the Ho^3+^ (3 mol%) doped SrZrO_3_ phosphor. Thus, this could be useful for w-LEDs because a CCT < 5000 K gives warm-white LEDs for home gadgets.

### 3.5 Judd–Ofelt intensity parameters from PLE and radiative properties

The familiar Judd–Ofelt intensity parameters (*Ω*_*λ*_ with *λ* = 2, 4, and 6) were adopted to figure out the fluorescence branching ratios, spontaneous emission probabilities, and radiative lifetimes of the excited multiplets to assess the undertaking of lasers and luminescent materials. As per Judd–Ofelt (J–O) theory,^49,50^ the J–O intensity parameters (*Ω*_*λ*_ with *λ* = 2, 4, and 6) can be examined conventionally from absorption spectra by evaluating the measured and computed spectral line strengths of the excited 4f–4f electronic transitions using least-square or chi-square fit methods. However, in recent decades,^42,51,52^ a simple approach has been proposed to evaluate J–O intensity parameters from the assessment of excitation spectra. This approach is successfully applied to Nd^3+^, Er^3+^ and Dy^3+^ doped various phosphor powders.

Normally, the excitation and absorption spectral difference lies in the intensity ratio of the fluorescence excitation to the absorption (*i.e.*, relative fluorescence quantum efficiency). Therefore, the excitation and absorption spectra will coincide exactly while relative fluorescence quantum efficiency maintains to be constant at different wavelengths. Once the experimental excitation spectrum is corrected to the corresponding absorption spectrum in which the excited states are followed by a very fast non-radiative relaxation to the monitored level.^53,54^ In this work, the excited multiplets of Ho^3+^: ^5^G_5_, ^5^F_2_(^3^K_8_), ^5^G_6_, ^5^F_3_ levels are nonradiatively relaxed to ^5^F_4_ + ^5^S_2_ monitored level may satisfy the above statement, and excited multiplets chosen as ideal ones for the determination Judd–Ofelt parameters. The calculated and measured relative excited line strength for the attended electric-dipole transitions across the *aJ* and *bJ*′ levels are determined using the following expressions,^55,56^6
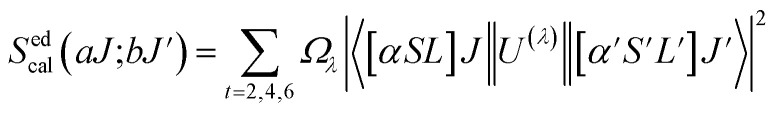
7
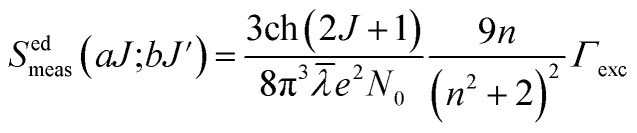
where *Ω*_*λ*_ is the J–O intensity parameter, which is used in the environmental field effect of intermixing states, *i.e.*, 4f^*N*−1^5d and 4f^*N*−1^5g. *U*(*λ*) is the doubly reduced matrix tensor operator and it is found in the coupling approximations approach, which is taken from the literature.^56^ The term *n* ∼2.12 is the refractive index of SrZrO_3_ material.^57^ The average wavelength of the excitation band is denoted as *λ̄*, *N*_0_ is the ion concentration and *Λ̄* is the integrated relative excitation intensity of each band. *Ω*_*λ*_ parameters were predicted by a least square fitting technique.^43^Table 2 shows relative spectral line strengths of excited transitions for Ho^3+^ ions (3 mol%) in the SrZrO_3_ phosphor.^55^ The root average square deviation (*δ*_rms_) between experimental line strengths is,8
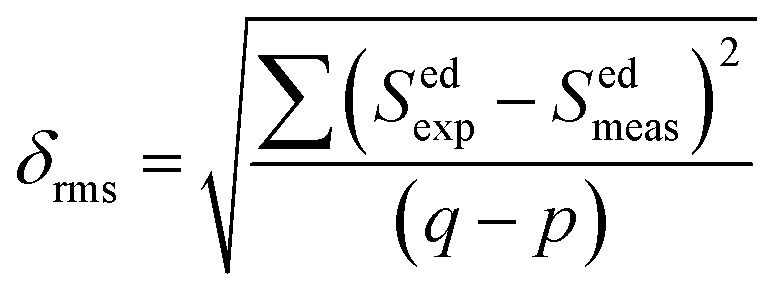
where *q* and *p* are fitting parameters as transition number, and it has been used in our case as *q* = 8 and *p* = 3 in the best least square fitting procedure. The observed small *δ*_rms_ value (see Table 2) is indicative of the validity and fit quality in J–O theory.

**Table 2:** Doubly reduced matrix elements ‖*U*^2^‖^*λ*^ (*λ* = 2, 4 and 6), relative line strengths (*S*^ed^) (×10^−20^ cm^2^) for the observed excitation/absorption bands of Ho^3+^ doped various host matrices

Transitions from ^5^I_8_ →	Excitation band wavenumber (*ν* cm^−1^)	‖*U*^2^‖^2^	‖*U*^2^‖^4^	‖*U*^2^‖^6^	aSrZrO_3_:0.03Ho^3+^		YLiF^55^	GdLiF_4_ (ref. 55)	LuLiF_4_ (ref. 55)
					*S* ^ed^_meas_	*S* ^ed^_cal_	*S* ^ed^_meas_	*S* ^ed^_meas_	*S* ^ed^_meas_
^3^K_6_ + ^3^F_4_	29 922	0.0026	0.1263	0.0073	0.101	0.024	0.309	0.341	0.251
^3^L_9_ + ^5^G_3_	28 902	0.0185	0.0052	0.1169	0.080	0.031	0.394	0.379	0.300
^3^H_6_ + ^3^H_5_	27 685	0.254	0.2337	0.1609	0.059	0.173	1.310	4.377	1.276
^5^G_4_ + ^3^K_7_	25 846	0.0058	0.0361	0.0697	0.012	0.021	0.325	0.345	0.307
^5^G_5_	23 872	0	0.5338	0.0002	0.087	0.085	1.161	1.199	1.120
^5^G_6_	22 020	1.5201	0.8410	0.1535	0.809	0.791	3.893	4.177	4.014
^5^F_2_ + ^3^K_8_	21 186	0.0208	0.0334	0.3576	0.094	0.083	0.392	0.446	0.248
^5^F_3_	20 542	0	0	0.3464	0.084	0.067	0.716	0.811	0.375
*δ*_rms_					± 0.011		± 0.117	± 0.114	± 0.108

a Present work.

Table 3 describes the J–O intensity parameters considering various host matrices.^58–62^ The *Ω*_2_ parameter indicates ionicity (or covalence) of RE-O bonds and is related to the local structure. *Ω*_4_ and *Ω*_6_ are non-sensitive to the dependence structure and are attributed to the stiffness of the host; however, the various active ions alter the characteristics of the evaluated spontaneous emission transitions. For example, from Table 3, the Ho^3+^ doped SrZrO_3_ phosphor shows a lower ionic nature between Ho–O bonds compared with other oxide-based host matrices. On the other hand, when compared with a fluoride-based host, the Ho^3+^ doped SrZrO_3_ phosphor shows a higher ionic nature between the Ho–O bonds because of the lesser values of the J–O intensity parameter, *Ω*_2_.

**Table 3:** Relative Judd–Ofelt intensity parameters, (*Ω*_*λ*_ ×10^−20^ cm^2^, *λ* = 2, 4 and 6) of various host matrices

Host	*Ω*_2_	*Ω*_4_	*Ω*_6_	Order
aSrZrO_3_:0.03Ho^3+^	0.41	0.16	0.19	*Ω*_6_ > *Ω*_4_ > *Ω*_2_
Y_3_Al_5_O_15_(YAG)^58^	0.04	2.67	1.89	*Ω*_4_ > *Ω*_6_ > *Ω*_2_
Lu_3_Al_5_O_12_ (ref. 59)	0.17	2.08	1.92	*Ω*_4_ > *Ω*_6_ > *Ω*_2_
LaF_3_ (ref. 60)	1.16	1.38	0.88	*Ω*_2_ > *Ω*_4_ > *Ω*_6_
LiYF_4_ (ref. 61)	1.01	1.71	1.21	*Ω*_4_ > *Ω*_6_ > *Ω*_2_
LiYF_4_ (ref. 62)	0.96	2.05	1.43	*Ω*_4_ > *Ω*_6_ > *Ω*_2_

a Present work.

## 4. Conclusions

The SrZrO_3_:Ho^3+^ phosphors were produced by a sol–gel system and were analyzed by X-ray diffraction, FTIR, UV-visible and photoluminescence spectroscopic techniques. We have identified absorption bands around 365, 421, 454, 468, 489, 542, and 645 nm from the UV-visible diffuse reflectance spectrum of the 4f-4f configuration of Ho^3+^ transitions. The optical band gaps (*E*_opt_) were found to be 5.21 eV (direct transition), 5.43 eV (indirect transition), respectively for the Ho^3+^ doped SrZrO_3_ phosphor. Upon 454 nm excitation, the Ho^3+^ doped SrZrO_3_ phosphor exhibits high green and low red emission bands that are connected to the respective ^5^F_4_ + ^5^S_2_ → ^5^I_8_ (545 nm) and ^5^F_5_ → ^5^I_8_ (652 nm) transitions of Ho^3+^ ions. The fluorescence quenching of the studied phosphor samples was evaluated by looking at the critical distance between Ho–Ho ions as well as the strength of dipole–dipole (d–d), dipole–quadruple (d–q) and quadruple–quadruple (q–q) interactions. The obtained CCT was 5654 K for the optimum concentration of Ho^3+^ (3 mol%) doped in the SrZrO_3_ phosphor suggesting that it may be useful for w-LEDs. In addition, using excitation spectrum of the optimized phosphor, the Judd–Ofelt intensity parameters (*Ω*_*λ*_ with *λ* = 2, 4 and 6) for Ho^3+^ were estimated and compared with other hosts. The observed results of photoluminescence properties suggested that the SrZrO_3_:0.03Ho^3+^ phosphor may be advantageous for green emitting optoelectronic applications.

## Conflicts of interest

There are no conflicts to declare.

## Supplementary Material
